# Environmental Conditions Modulate the Protein Content and Immunomodulatory Activity of Extracellular Vesicles Produced by the Probiotic Propionibacterium freudenreichii

**DOI:** 10.1128/AEM.02263-20

**Published:** 2021-01-29

**Authors:** Vinícius de Rezende Rodovalho, Brenda Silva Rosa da Luz, Aurélie Nicolas, Fillipe Luiz Rosa do Carmo, Julien Jardin, Valérie Briard-Bion, Gwénaël Jan, Yves Le Loir, Vasco Ariston de Carvalho Azevedo, Eric Guédon

**Affiliations:** a INRAE, Institut Agro, STLO, Rennes, France; b Laboratory of Cellular and Molecular Genetics, Institute of Biological Sciences, Federal University of Minas Gerais, Belo Horizonte, Brazil; University of Naples Federico II

**Keywords:** EV, NF-κB, anti-inflammatory, comparative proteomics, growth conditions, immunomodulation, membrane vesicle, protein-protein interactions

## Abstract

Propionibacterium freudenreichii is a probiotic Gram-positive bacterium with promising immunomodulatory properties. It modulates regulatory cytokines and mitigates the inflammatory response *in vitro* and *in vivo*. These properties were initially attributed to specific bacterial surface proteins. Recently, we showed that extracellular vesicles (EVs) produced by *P. freudenreichii* CIRM-BIA129 mimic the immunomodulatory features of parent cells *in vitro* (i.e., modulating NF-κB transcription factor activity and interleukin-8 release), which underlies the role of EVs as mediators of the probiotic effects of the bacterium. The modulation of EV properties, and particularly of those with potential therapeutic applications, such as the EVs produced by the probiotic *P. freudenreichii*, is one of the challenges in the field to achieve efficient yields with the desired optimal functionality. Here, we evaluated whether the culture medium in which the bacteria are grown could be used as a lever to modulate the protein content and, hence, the properties of *P. freudenreichii* CIRM-BIA129 EVs. The physical, biochemical, and functional properties of EVs produced from cells cultivated on laboratory yeast extract lactate (YEL) medium and cow milk ultrafiltrate (UF) medium were compared. UF-derived EVs were more abundant and smaller in diameter, and they displayed more intense anti-inflammatory activity than YEL-derived EVs. Furthermore, the growth media modulated EV content in terms of both the identities and abundances of their protein cargos, suggesting different patterns of interaction with the host. Proteins involved in amino acid metabolism and central carbon metabolism were modulated, as were the key surface proteins mediating host-propionibacterium interactions.

**IMPORTANCE** Extracellular vesicles (EVs) are cellular membrane-derived nanosized particles that are produced by most cells in all three kingdoms of life. They play a pivotal role in cell-cell communication through their ability to transport bioactive molecules from donor to recipient cells. Bacterial EVs are important factors in host-microbe interactions. Recently, we have shown that EVs produced by the probiotic *P. freudenreichii* exhibited immunomodulatory properties. We evaluate here the impact of environmental conditions, notably culture media, on *P. freudenreichii* EV production and function. We show that EVs display considerable differences in protein cargo and immunomodulation depending on the culture medium used. This work offers new perspectives for the development of probiotic EV-based molecular delivery systems and reinforces the optimization of growth conditions as a tool to modulate the potential therapeutic applications of EVs.

## Abstract (precis)

Extracellular vesicles (EVs) are cellular membrane-derived nanosized particles that are produced by most cells in all three kingdoms of life. They play a pivotal role in cell-cell communication through their ability to transport bioactive molecules from donor to recipient cells.

## INTRODUCTION

Probiotic organisms are increasingly being used in medical and technological contexts because of their health benefits (1, 2). Among these organisms, dairy bacteria such as Propionibacterium freudenreichii are of particular value because of their long-term safe consumption and economic interest (3, 4). *P. freudenreichii* is a Gram-positive bacterium that is used traditionally as a Swiss-type cheese starter (5) but has also been studied with respect to the production of vitamin B_12_ (6, 7) and organic acids (8, 9). Furthermore, this bacterium can survive for a considerable period *in vitro* and under cheese-making conditions (10). It can also survive and adapt metabolically to animal and human gastrointestinal tracts (11–13) and has been identified in the fecal samples from a discrete cohort of human preterm breastfed infants (14, 15).

*P. freudenreichii* has long been studied for its probiotic properties, such as modulating the composition of the microbiota, antitumor activity, and immunomodulation (3). This species mainly modulates the composition of the microbiota through a bifidogenic effect, promoted by abundantly produced metabolites such as 1,4-dihydroxy-2-naphthoic acid (DHNA) and 2-amino-3-carboxy-1,4-naphthoquinone (ACNQ) (16–19). Its antitumor activity is linked to the production of short-chain fatty acids such as propionate and acetate (20–24). Regarding immunomodulation, some proteins in this species, and particularly surface layer protein B (SlpB), have been associated with a reduction in the proinflammatory cytokines released and/or an increase in the release of anti-inflammatory cytokines both *in vitro* and *in vivo* (19, 25–29).

In addition to bacterial surface exposure, we recently showed that some immunomodulatory *P. freudenreichii* proteins are found associated with extracellular vesicles (EVs) (30). EVs are spherical nanometric structures produced by cells in the three domains of life (31). They are composed of lipid bilayers and an internal functional cargo, implicated in several biological processes that include interactions with host cells (31–34). The production of EVs has been demonstrated in several probiotic bacteria species (35–41). In the case of *P. freudenreichii*, EVs produced by the CIRM-BIA129 strain had a proteome predicted to interact with key protein components of the human inflammatory response, which was reinforced by the ability of EVs to reduce both the activity of the transcription factor NF-κB and interleukin-8 (IL-8) release in an intestinal epithelial cell model (30). The modulation exerted by ultrafiltrate (UF)-derived EVs occurred when the inflammatory response was induced by bacterial lipopolysaccharide (LPS) but not by other inducers, such as tumor necrosis factor alpha (TNF-α) and IL-1β. In addition, the EV-borne SlpB protein was shown to play a role in this immunomodulation, because EVs secreted by a Δ*slpB* mutant displayed reduced anti-inflammatory activity (30).

As well as their functional role in modulating interactions with the host, bacterial EVs are themselves modulated by specific conditions. In other words, environmental shifts impact the production and functional properties of EVs (42). For example, exposure to antibiotics induces vesiculation in several Staphylococcus aureus strains (43) and modifies the protein content of EVs secreted by Acinetobacter baumannii DU202 (44) and Campylobacter jejuni 81-176 (45). Iron-limiting conditions were shown to be associated with quantitative changes to the EV proteome of pathogenic and probiotic Escherichia coli strains (46, 47), of Helicobacter pylori 60190 (48), and of Mycobacterium tuberculosis H37Rv (49). Salt stress was also shown to be linked to drastic changes in the protein content and proinflammatory activity of EVs secreted by Listeria monocytogenes 10403S (50, 51). The EV proteome or its activity also changed in line with growth phases in Bacillus subtilis 168 (52), Rhizobium etli CE3 (53), Pseudomonas aeruginosa PAO1 (54), and H. pylori 26695 (55) and with the composition of media in Gallibacterium anatis 12656-12 (56) and Pseudomonas putida KT2440 (57). The bacterial properties of EVs were also affected by pH shifts (58) and exposure to epibromohydrin (59) or cannabidiol (60) and to other environmental conditions (61).

The impact of environmental conditions on EV production and function still remains poorly documented in probiotic bacteria, although it may represent a tool to modulate the properties of EVs and have potential therapeutic applications. Therefore, our aim was to investigate whether different growth conditions impact the production of *P. freudenreichii*-derived EVs as well as modulate their properties. In the present study, we have shown that *P. freudenreichii* CIRM-BIA129 cultured in milk UF or yeast extract-lactate (YEL) growth medium produced EVs with distinct physicochemical, biochemical, and functional properties. In particular, UF- and YEL-derived EVs displayed considerable differences in protein cargo and immunomodulation. This study is a comparative analysis of the properties of the EVs produced by *P. freudenreichii* under various growth conditions and will contribute to understanding how probiotic traits are affected by different contexts. It also suggests interventional opportunities for the engineering of EV content and activity, enabling improvements to their potential technological and therapeutic roles.

## RESULTS

### The production of EVs by *P. freudenreichii* is dependent on growth conditions.

**FIG 1:**
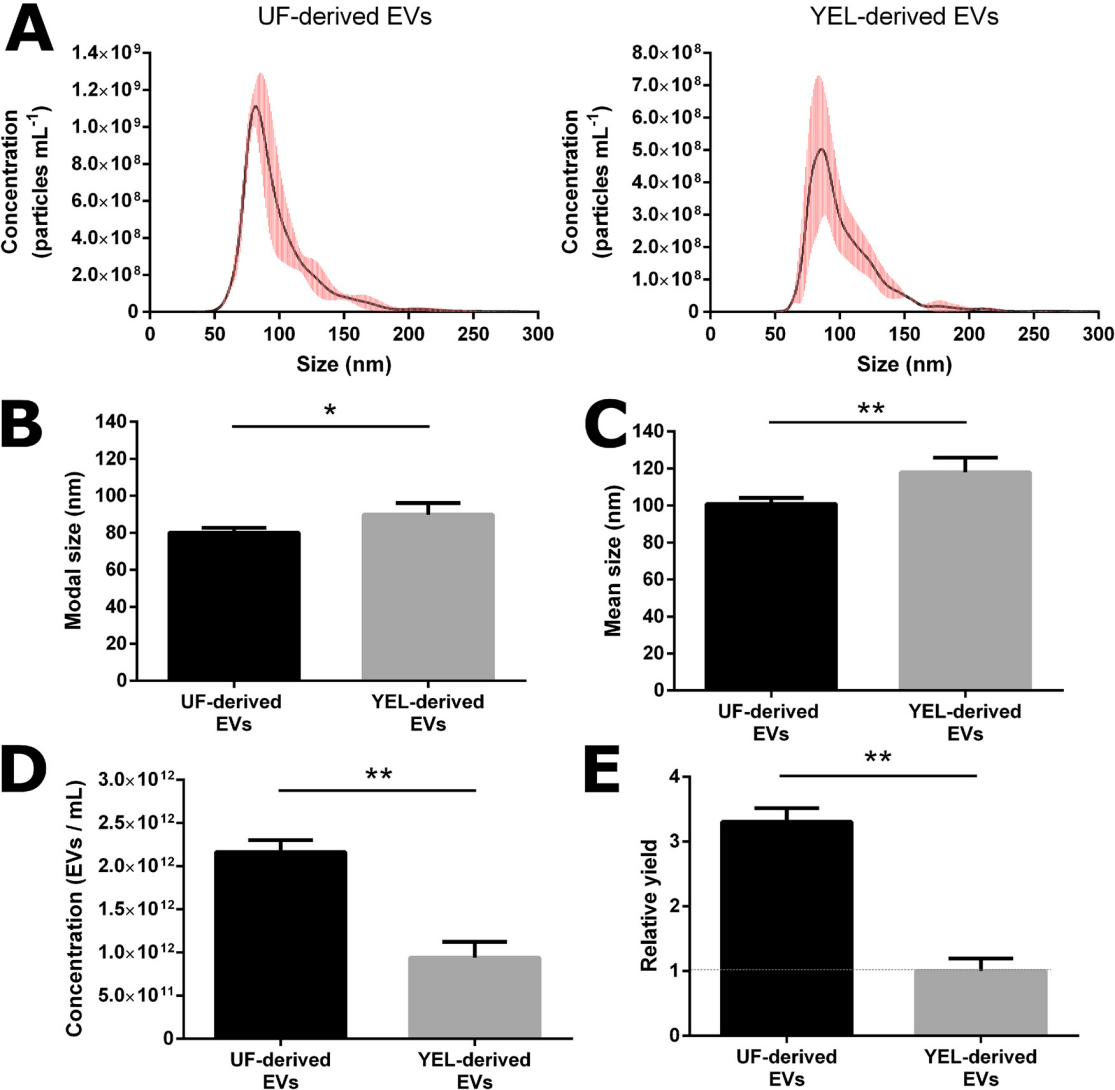
Characteristics of *P. freudenreichii*-secreted EVs under different growth conditions. (A) Size distribution of EVs derived from UF (left) and YEL (right) culture media. (B and C) Mode (B) and mean (C) diametric sizes of EVs produced from UF and YEL culture media. (D) Total concentrations of EVs produced from UF and YEL culture media. (E) Relative yields of EVs (normalized by the amount of bacterial cells in each culture medium). Size and concentration measurements were acquired using nanoparticle tracking analysis (NTA). Data are expressed as means ± standard deviations of values obtained from at least three independent biological replicates. Asterisks indicate statistical significance as evaluated by the Mann-Whitney test: **, *P* ≤ 0.01; *, *P* ≤ 0.05.

During this work, we tried to determine whether environmental conditions, i.e., different growth media, affected the production of EVs by *P. freudenreichii* CIRM-BIA129. For that purpose, the sizes and concentrations of EVs purified from UF and YEL culture media were evaluated. Both conditions yielded EVs with a monodisperse size distribution, although YEL-derived EVs were less abundant than UF-derived EVs (Fig. 1A). They also displayed differences in diameter: UF-derived EVs had a modal size of 80.06 ± 2.606 nm and a mean size of 100.9 ± 3.204 nm, whereas YEL-derived EVs were significantly larger (*P* < 0.05), with a modal size of 89.72 ± 6.324 nm and a mean size of 117.9 ± 7.856 nm (Fig. 1B and C). The total concentration of EVs was significantly higher (*P* < 0.01) from UF (2.164 × 10^12^ ± 1.383 × 10^11^ EVs ml^−1^) than from YEL (9.394 × 10^11^ ± 1.846 × 10^11^ EVs ml^−1^) medium (Fig. 1D). Finally, the EV relative yield of CIRM-BIA129 (i.e., the amount of recovered EVs normalized by the amount of bacterial cells at sampling time) was more than three times higher from UF than from YEL medium (Fig. 1E).

### The biological activity of *P. freudenreichii*-derived EVs is dependent on growth conditions.

**FIG 2:**
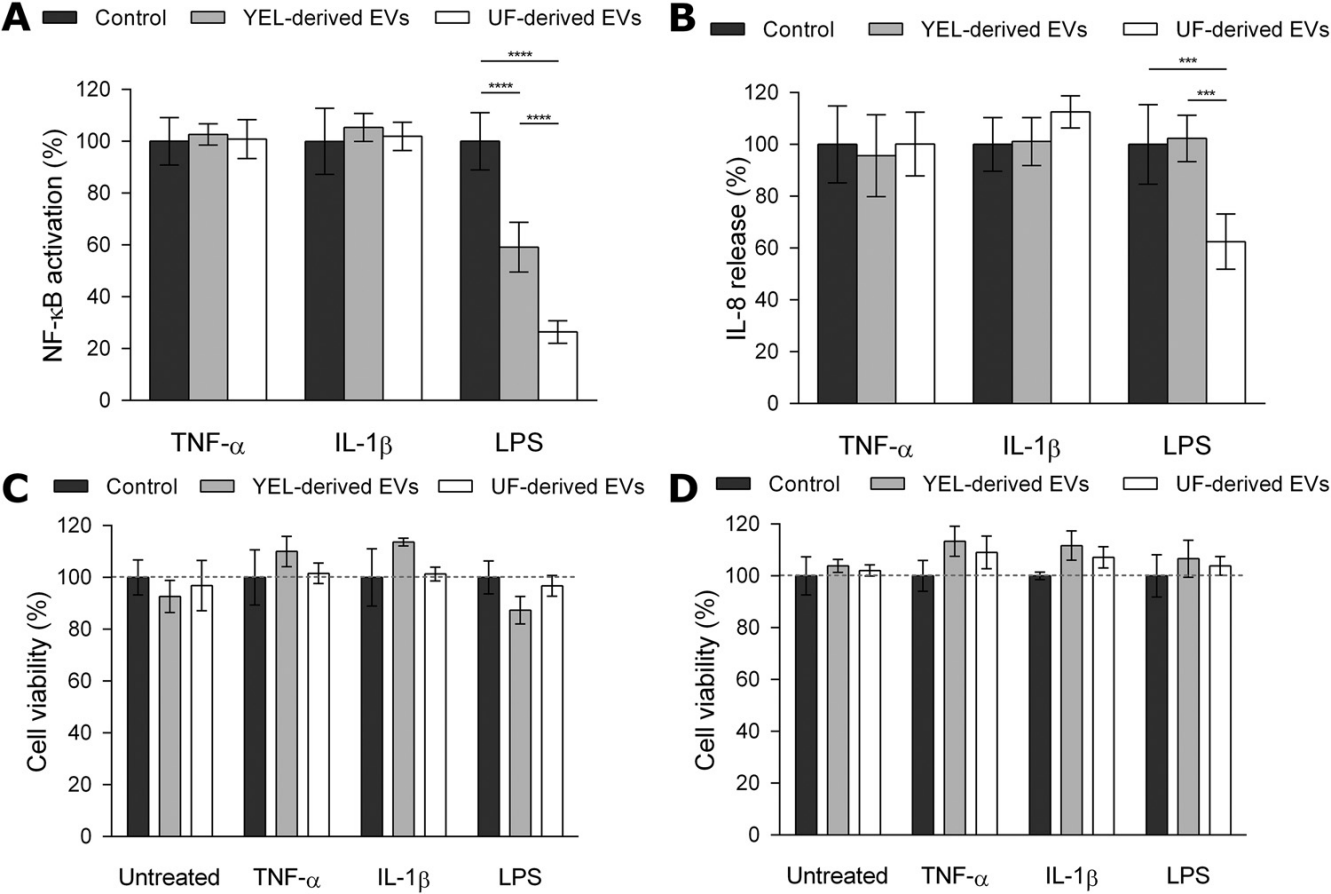
*P. freudenreichii*-secreted EVs play different biological roles depending on growth conditions. (A) Percent NF-κB transcription factor activity in HT-29/kb-seap-25 cells left untreated or treated with the inflammatory inducer TNF-α (1 ng ml^−1^), IL-1β (1 ng ml^−1^), or LPS (1 ng ml^−1^) in the presence or absence of UF- and YEL-derived EVs (1.0 × 10^9^ EVs ml^−1^). The values are normalized by the control conditions (control TBS buffer or stimulation by the inducer in the absence of EVs). (B) Percentage of IL-8 released by HT-29 intestinal epithelial cells after stimulation by LPS (1 ng ml^−1^), TNF-α (1 ng ml^−1^), or IL-1β (1 ng ml^−1^) inducer in the presence or absence of UF- and YEL-derived EVs (1.0 × 10^9^ EVs ml^−1^). The values are normalized to the control conditions (control TBS buffer or stimulation by inducer in the absence of EVs). (C and D) Percent viability of HT-29/kb-seap-25 reporter (left) and HT-29 parental (right) cells before or after stimulation by LPS (1 ng ml^−1^), TNF-α (1 ng ml^−1^), or IL-1β (1 ng ml^−1^) inducer in the presence or absence of UF- and YEL-derived EVs (1.0 × 10^9^ EVs ml^−1^). The values are normalized by the control conditions (control TBS buffer or stimulation by an inducer in the absence of EVs). Data are expressed as mean ± standard deviation of values obtained from at least three independent biological replicates. Asterisks indicate statistical significance as evaluated by two-way ANOVA with Tukey's multiple-comparison test: ****, *P* ≤ 0.0001; ***, *P* ≤ 0.001.

EVs derived from the two growth conditions were also evaluated in terms of their biological activity *in vitro*, i.e., NF-κB activation and IL-8 release. UF-derived *P. freudenreichii* CIRM-BIA129 EVs had previously been shown to exert an immunomodulatory effect on HT-29 human intestinal epithelial cells via the NF-κB pathway, i.e., modulation of transcription factor NF-κB activity and IL-8 release (30). To determine whether growth conditions also modulate EV activity, we compared the ability of the EVs produced from UF and YEL media to modulate the regulatory activity of the NF-κB transcription factor and the release of IL-8 from human intestinal epithelial cells. For that purpose, NF-κB/SEAP HT-29 cells, which are designed to measure NF-κB regulatory activity (62), and HT-29 parental cells were induced to an inflammatory state by different proinflammatory inducers and treated with EV preparations. Whatever the growth medium, EVs exerted an inhibitory effect on the regulatory activity of the NF-κB transcription factor only when the inflammation pathway was induced by LPS (Fig. 2A). Nevertheless, EV-mediated reduction of the LPS-induced proinflammatory effect was growth medium dependent. Indeed, the anti-inflammatory activity of EVs was significantly more intense with UF-derived than with YEL-derived EVs (*P* < 0.0001) (Fig. 2A). The specific LPS-induced anti-inflammatory effect of EVs was confirmed by the evaluation of IL-8 release from the HT-29 parental cell line. However, in this case, only UF-derived EVs were able to reduce LPS-induced IL-8 release (Fig. 2B). Finally, UF- and YEL-derived EVs had no cytotoxic effect on the two HT-29 cell lines, indicating that reductions in NF-κB activation and IL-8 release were not associated with cell death (Fig. 2C and D).

### EVs produced under different growth conditions contain shared and exclusive proteins.

**FIG 3:**
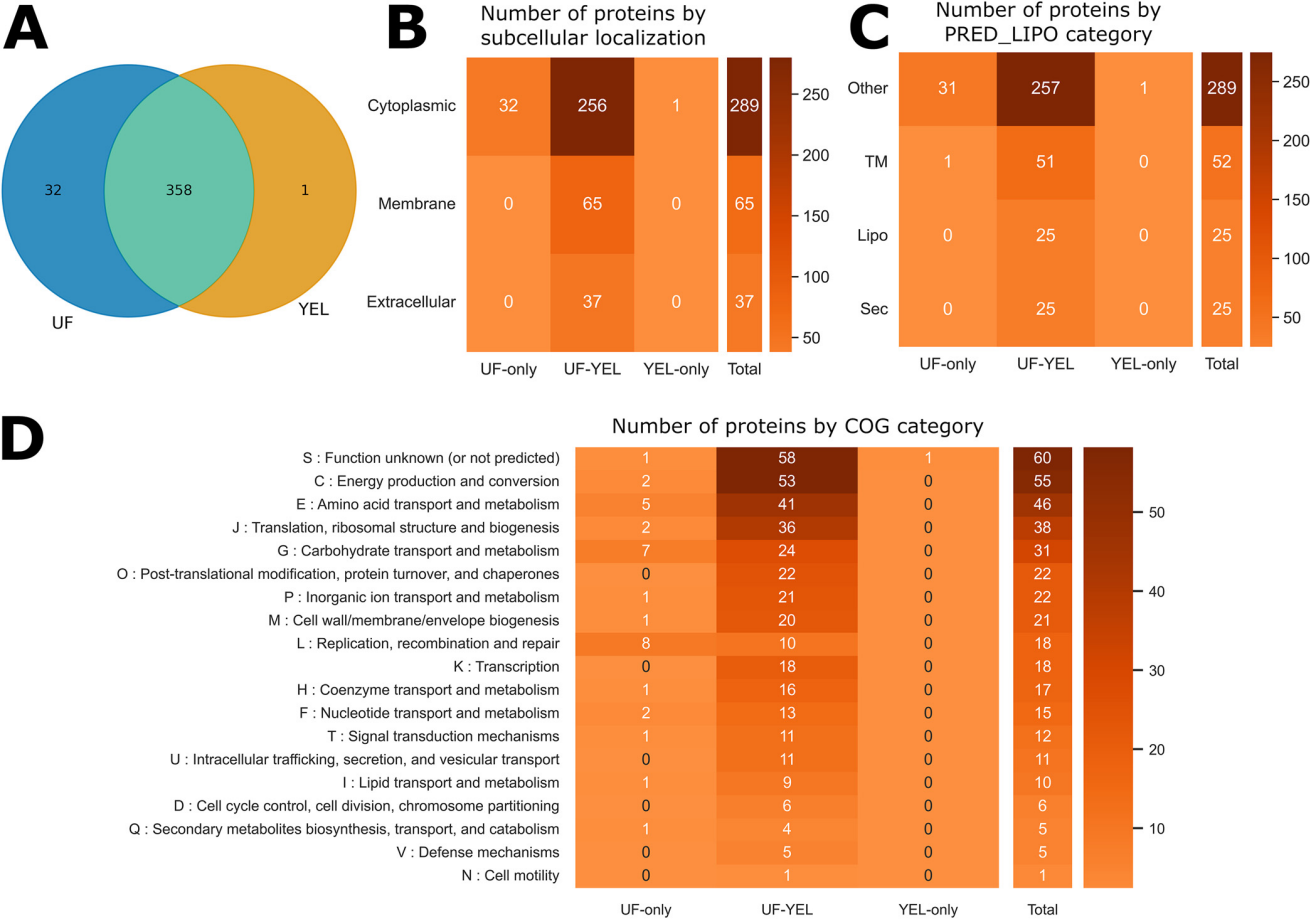
Qualitative proteomics can reveal differences in EV protein content as a function of growth conditions. (A) Venn diagram showing the number of shared and specific proteins for EVs under the two growth conditions. (B to D) Heatmaps showing the distribution of proteins as a function of growth condition categories and functional predictions. Columns show growth condition categories (UF-only, proteins identified exclusively in UF-derived EVs; YEL-only, proteins identified exclusively in YEL-derived EVs; UF-YEL, proteins identified under both growth conditions). Rows show subcellular localization prediction (cytoplasmic, membrane, or extracellular) (B), PRED-LIPO prediction (Sec, secretion signal peptide; Lipo, lipoprotein signal peptide; TM, transmembrane; Other, no signals found) (C), and COG functional categories (D).

To identify potential factors involved in the differences in biological activity between UF- and YEL-derived EVs, we characterized the protein content of EVs produced under the two growth conditions (see Table S4 in the supplemental material). Qualitative proteomics analysis enabled the identification of 391 proteins, 32 of which were exclusively detected in UF-derived EVs, one was exclusively detected in YEL-derived EVs, and 358 were common to both growth conditions (Fig. 3A). Concerning subcellular localization, all exclusive proteins (UF and YEL exclusive) were predicted to be cytoplasmic. The shared proteins were mainly cytoplasmic (*n* = 256), but some were also predicted to be membrane proteins (*n* = 65) or extracellular proteins (*n* = 37) (Fig. 3B). Predicted lipoproteins were only identified among proteins found under both conditions (*n* = 25) (Fig. 3C). The frequencies of Clusters of Orthologous Groups (COG) categories were well distributed among shared proteins, mainly being related to metabolism and information processing. UF-exclusive proteins were mainly linked to carbohydrate or amino acid metabolism and DNA processing, while the only YEL-exclusive protein had an unknown function (Fig. 3D).

### A comparison of growth conditions reveals several differentially abundant EV proteins.

**FIG 4:**
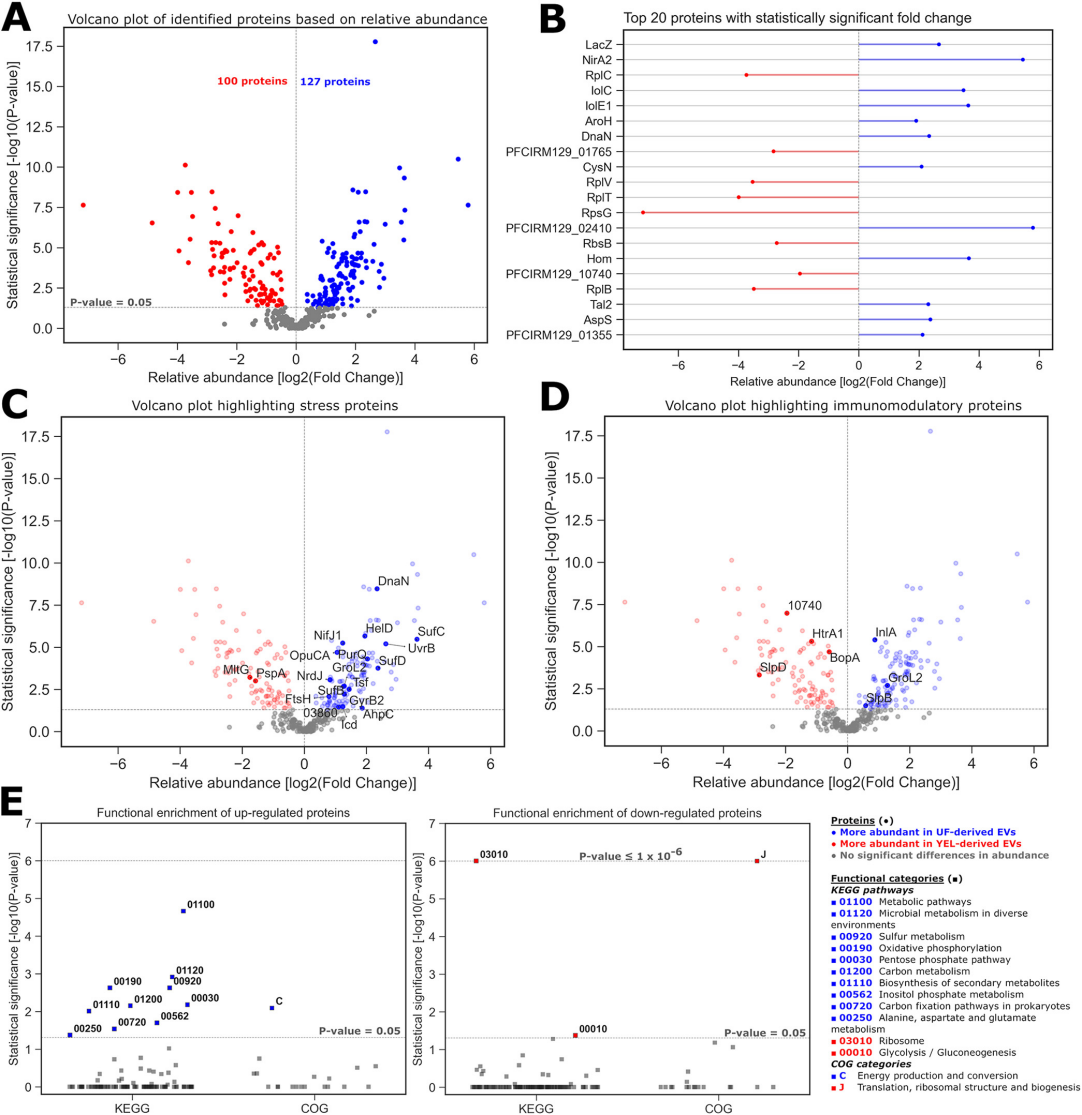
EVs derived from different growth conditions carry proteins with different relative abundances. (A) Volcano plot showing the relative abundance of proteins from UF-derived EVs versus YEL-derived EVs (fold change UF/YEL) as well as the significance of their fold changes. The results are presented according to a logarithmic scale, with positive fold change ratios shown in blue, negative fold change ratios shown in red, and nonsignificant ratios shown in gray. The horizontal dotted line shows the threshold of significance, and the vertical line indicates a logarithmic fold change of zero. (B) Relative abundance of proteins with the highest significant UF/YEL fold changes. (C and D) Volcano plots highlighting subsets of proteins reportedly associated with a stress response (C) or immunomodulation (D) in *P. freudenreichii* or other bacterial strains. (E) Enrichment analysis of KEGG pathways and COG categories for the upregulated (left) and downregulated (right) proteins. Significant results are shown in blue for categories enriched in upregulated proteins, in red for categories enriched in downregulated proteins, and in gray for categories that are not statistically enriched. The lower dotted line represents the minimal threshold of significance, and the higher dotted line indicates a cap for categories with *P* values of 1 × 10^−6^ or lower.

Qualitative proteomic analysis pinpointed proteins specific to YEL- and UF-derived EVs. Further, to better grasp the impact of growth conditions on EV content, the relative abundance of the proteins was analyzed under each condition. Quantitative proteomics showed that the relative abundance of EV proteins could vary as a function of culture medium (Fig. S1). Among the total of 391 identified proteins, the abundances of 164 proteins did not differ between YEL- and UF-derived EVs (*P* > 0.05), 127 proteins were significantly more abundant in UF-derived EVs (*P* ≤ 0.05, log[fold change] > 0), and 100 proteins were significantly more abundant in YEL-derived EVs (*P* ≤ 0.05, log[fold change] < 0) (Fig. 4A). Among the proteins more abundant in UF-derived EVs, the most significant included those related to carbohydrate (LacZ, IolC, and IolE1), amino acid (AroH and Hom), and energy (NirA2) metabolism. Among the proteins more abundant in YEL-derived EVs, the most significant were mainly ribosomal proteins (RplC, RplV, RplT, RplB, and RpsG). Differentially abundant proteins under each condition also included those of unknown function or poorly characterized (PFCIRM129_01355, PFCIRM129_01765, PFCIRM129_02410, and PFCIRM129_10740) (Fig. 4B). Interestingly, among EV proteins undergoing significant changes to their abundance as a function of the culture medium, we identified several that had reportedly been associated with a stress response (Fig. 4C) or immunomodulatory properties (Fig. 4D) in *P. freudenreichii* or other bacterial species. Functional enrichment analysis (Tables S5 and S6) showed that proteins that were more abundant in UF-derived EVs were mainly related to energy, carbohydrate, amino acid, and sulfur metabolism KEGG pathways, together with COG category C (energy production and conversion). On the other hand, proteins more abundant in YEL-derived EVs were mainly related to glycolysis/gluconeogenesis and ribosome KEGG pathways, together with COG category J (translation, ribosomal structure, and biogenesis) (Fig. 4E).

### Predictions of interactions highlight the relevant bacterial proteins potentially implicated in immunomodulation.

**FIG 5:**
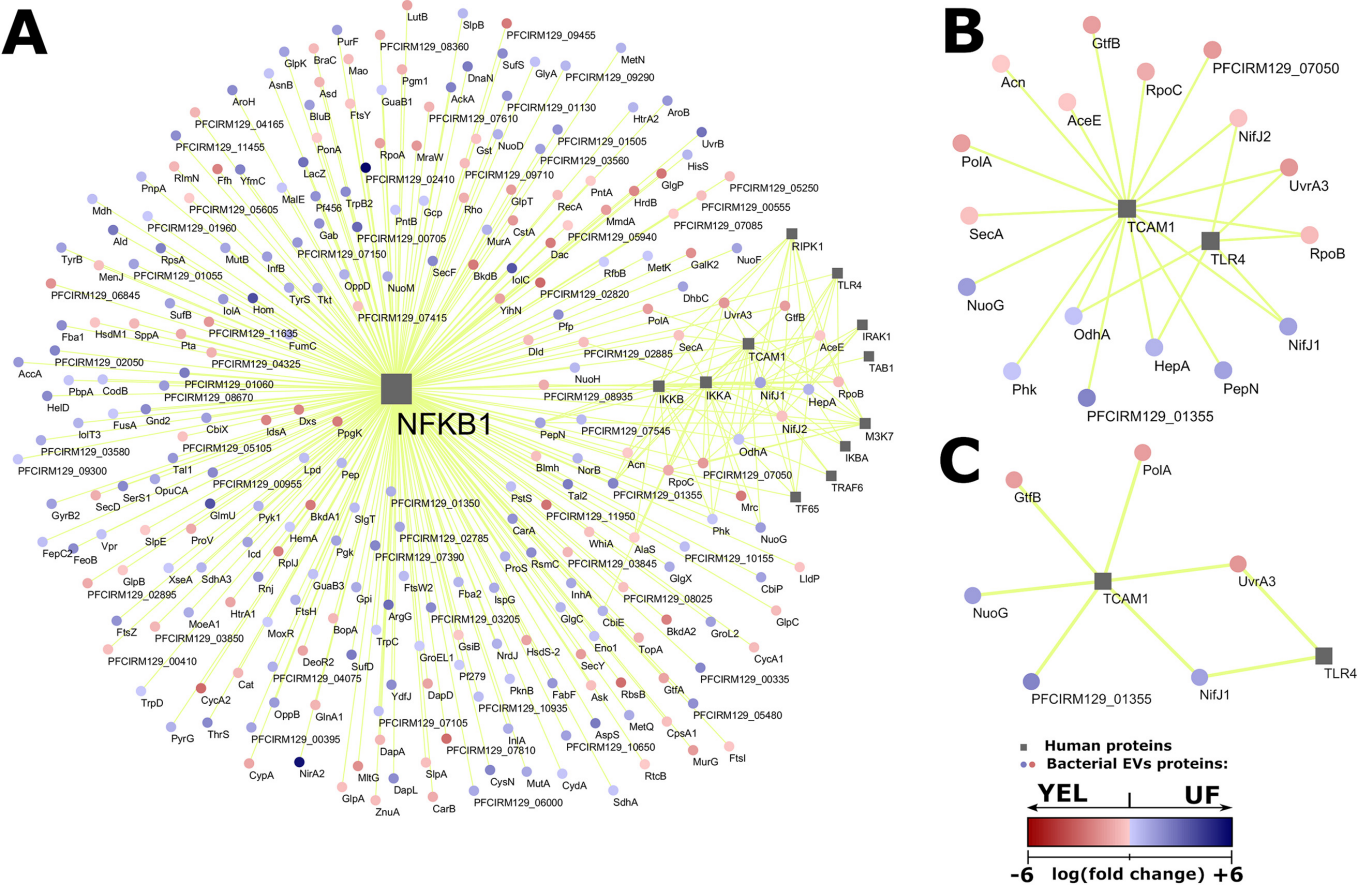
Prediction of protein-protein interactions highlights relevant bacterial proteins. (A to C) A network representation of predicted interactions filtered by human proteins that correspond to the LPS-induced part of the NF-κB pathway (A), by uman proteins involved in the LPS-induced NF-κB pathway but not in induction by TNF-α or IL-1β (B), and by bacterial proteins whose abundance was considered significant by quantitative proteomic analysis (C). Human proteins are represented by gray squares, and bacterial proteins are represented by red or blue circles. Predictions of interactions are represented by yellow lines connecting squares and circles. The relative abundance of bacterial proteins is seen according to the scale in the bottom right corner (logarithm of fold change), where positive values (blue) indicate a higher abundance in UF-derived EVs and negative values (red) indicate a higher abundance in YEL-derived EVs.

The qualitative and quantitative differences in the protein content of EVs revealed factors that might vary with growth conditions. Our next aim was to investigate whether this variation, when translated into different patterns of interaction, might offer insights into the mechanisms underlying the differential activity of EVs. Therefore, 101 human proteins from the NF-κB pathway and 391 bacterial proteins found in EVs were submitted to the prediction of protein-protein interactions. A total of 893 interactions were considered to be valid (minimum score of 0.9765), involving 49 human and 266 bacterial proteins (Table S7). Next, we performed progressive filtering procedures to select meaningful interactions according to experimental criteria. First, in view of the fact that the anti-inflammatory activity of EVs was LPS specific, we filtered out interactions involving human proteins that were not related to the LPS-induced part of the NF-κB pathway, which resulted in a subnetwork of 357 interactions (Fig. 5A). A further filtering was then performed to retain only human proteins that were specific to the LPS-induced NF-κB pathway, i.e., not related to TNF-α or IL-1β induction. This resulted in 23 interactions, involving just two human proteins: TCAM1 and TLR4 (Fig. 5B). A final filtering was performed in bacterial proteins to retain only those whose differential expression was considered significant (*P* ≤ 0.05) according to quantitative proteomics (Fig. 5C). Therefore, TLR4 and TCAM1 human proteins were identified within the NF-κB signaling pathway as potential targets for immunomodulation. Moreover, novel bacterial proteins were suggested to be responsible for the differential intensity of the anti-inflammatory response resulting from various growth conditions: pyruvate synthase/pyruvate-flavodoxin oxidoreductase (NifJ1), the NADH-quinone oxidoreductase chain G (NuoG), an uncharacterized protein (PFCIRM129_01355), the GtfB glycosyltransferase (GtfB), a putative DNA polymerase I (PolA), and UvrABC system protein A (UvrA3).

## DISCUSSION

Several studies have demonstrated that the production, properties, and roles of bacterial EVs are affected by environmental conditions. Thus, monitoring the environmental conditions under which the bacteria are grown offers a tool to modulate the production and properties of EVs and may have some therapeutic applications (42). We recently reported the production of EVs by the probiotic *P. freudenreichii* CIRM-BIA129, and our study included their physicochemical and functional characterization (30). In particular, we showed that they displayed anti-inflammatory effects throughout modulation of the regulatory activity of the NF-κB transcription factor and IL-8 release in human epithelial cell models. However, whether the production and immunomodulatory effects of *P. freudenreichii*-secreted EVs could be modulated by environmental conditions still needed to be determined.

Here, we compared the properties of *P. freudenreichii* EVs purified from two growth media that are routinely used for *P. freudenreichii* cultures: cow’s milk ultrafiltrate (UF) medium and yeast extract-lactate (YEL) medium (29, 63, 64). YEL is the gold-standard laboratory medium for propionibacteria. This medium, in addition to yeast extract (providing the necessary growth factors) and sodium lactate (the preferred carbon and energy source), contains peptone (as a nitrogen source) (65). This medium was developed to mimic the growth conditions of propionibacteria in Swiss-type cheeses after fermentation of the cheese curd by lactic acid bacteria. We later developed a milk UF medium to mimic the growth of propionibacteria in fermented milk (63, 66). This medium represents the aqueous phase of cow milk, added with casein peptone and sodium lactate. UF and YEL were chosen to compare EV properties because they differentially impact the physiology of the bacterium, notably its growth parameters (biomass and generation time), the pH of the extracellular medium at the end of stationary phase, and cell viability after stress challenges (63, 64).

We demonstrated that culture media affect the physical, biochemical, and biological properties of *P. freudenreichii*-secreted EVs. Notably, compared to EVs recovered from YEL cultures, markedly more were recovered from UF cultures. In some bacterial species, an increase in EV production has been linked to an adaptation to stressful conditions (67–69). In the case of *P. freudenreichii*, UF could be considered a more stressful growth condition than YEL, since the bacterium accumulated higher proportions of trehalose during growth in UF than in YEL (64), with this sugar being involved in the response to stress, notably during acid adaptation and osmoadaptation (64, 70, 71). Moreover, some of the proteins that we found more abundant in UF than YEL-derived EVs were associated in the parent cells with the adaptation of the bacterium to stress (Fig. 4C). Among these, chaperonin GroL2 (GroL2), pyruvate synthase/pyruvate-flavodoxin oxidoreductase (NifJ1), and alkyl hydroperoxide reductase protein C22 (AhpC) were notably associated with the response to acid stress in this bacterium (72). Taken together, these results suggested that EV production in *P. freudenreichii* is affected by stress conditions and showed that the control of growth conditions might offer a lever to modulate EV production in this probiotic bacterium.

As mentioned above, in addition to impacting the physical properties of EVs, the culture medium also modulates EV content in terms of both the identities and abundances of its protein cargo. When the protein contents were compared, modest differences were observed between YEL- and UF-derived EVs. Indeed, more than 90% (*n* = 358) of EV proteins were found to be common to the YEL and UF media. Only one protein with an unknown function was specific to YEL, and 32 of the predicted cytoplasmic proteins were found to be exclusive to UF-derived EVs. This notably included several proteins with functions assigned to the COG categories of “replication, recombination, and repair” (L, *n* = 8), “carbohydrate transport and metabolism” (G, *n* = 7), and “amino acid transport and metabolism” (E, *n* = 5). The most important impact of the culture medium concerned the abundance of proteins packed into EVs. The abundance of approximately 60% of EV proteins changed significantly as a function of culture medium. In the case of YEL-derived EVs, the more abundant proteins were mainly related to ribosomes, glycolysis, and gluconeogenesis, which may have been linked to the higher growth rate of *P. freudenreichii* in YEL medium (63, 64). In contrast, UF-derived EVs were more enriched in proteins related to pentose phosphate, sulfur, secondary metabolites, inositol phosphate, amino acids, and energy metabolism, which are probably related to stress responses. Although the rules for the cargo selection and sorting into bacterial EVs remain elusive, especially in Gram-positive bacteria (73), it is often thought that the content of EVs reflects the physiology, metabolic status, and biological properties of the cells producing them (32, 52). Therefore, modulation of both the presence and abundance of proteins in EVs as a function of culture medium might be related to the level of their synthesis by cells. Indeed, it seems consistent that the relative abundance of a protein in a whole cell would affect its availability to be packed into EVs in the absence of, or in addition to, a selective process (34, 74). Accordingly, several proteins associated with the adaptation of the bacterium to UF or YEL medium were identified as being more abundant in UF- or YEL-derived EVs, respectively. For example, some of the proteins specifically present or found to be more abundant in UF-derived EVs were involved in the assimilation of the main carbon sources of UF, including beta-galactosidase (LacZ) (which hydrolyzes lactose into glucose and galactose) and inositol assimilation proteins (IolE1, IolC, IolT3, and IolA). However, one cannot exclude that, in addition to the level of synthesis by cells, some of these proteins are also purposely packed into EVs to perform specific functions, such as host cell interactions (75). To date, the molecular mechanisms that drive the recruitment of proteins into bacterial EVs have remained unclear. Determining which proteins are selected, and how, is of crucial value to identifying those purposely packed into EVs, notably to better understand their biological roles. Recently, we showed that abundance, charge, and subcellular localization could influence the protein availability of the vesicle cargo in S. aureus (74). Whether these rules are also applicable to the EVs secreted by *P. freudenreichii* still needs to be investigated, but our data may offer an additional opportunity to uncover the mechanisms governing the selection of proteins into EVs.

Finally, the culture medium in which bacteria are grown also modulates the biological activity of CIRM-BIA129 EVs. Indeed, UF- and YEL-derived EVs presented remarkable differences in terms of immunomodulation. We showed that EVs from both origins reduced the activity of NF-κB, a key mediator of inflammatory responses, but at different intensities. This reduction occurred specifically when inflammation was induced by bacterial LPS and was achieved in roughly 74% for UF-derived EVs but only 41% for YEL-derived EVs. This differential EV-mediated modulation of NF-κB activity has profound consequences on the cellular inflammatory response. Indeed, LPS-induced IL-8 release by HT-29 cells was significantly reduced by UF-derived EVs but not by YEL-derived EVs. To date, the bacterial effectors involved in the immunomodulatory activity of *P. freudenreichii*-secreted EVs are still unknown. We recently showed that SlpB participates in EV-mediated anti-inflammatory effects *in vitro*, since EVs derived from a Δ*slpB* mutant displayed a partial reduction of NF-κB activation compared to wild-type-derived EVs (30). However, it is not known whether the role of SlpB is direct or indirect. Among *P. freudenreichii*-associated immunomodulatory proteins, SlpB, internaline A (InlA), and the chaperonin GroL2 were more abundant in UF- than in YEL-derived EVs. In contrast, surface-layer protein D (SlpD), the solute binding protein of the ABC transport system (BopA), trypsin-like serine protease (HtrA1), and a protein of unknown function, PFCIRM129_10740, were more abundant in YEL-derived EVs. The differences in abundance of at least one of these recognized immunomodulatory proteins may explain the differential anti-inflammatory activity seen for UF- and YEL-derived EVs (27–29, 76).

One can also suppose that other differentially abundant proteins are implicated in the differential activity of EVs triggered by culture media. These include those bacterial proteins predicted to interact with the human proteins Toll-like receptor 4 (TLR4) and TIR domain-containing adapter molecule 1 (TICAM-1), which are of particular interest. Indeed, these two proteins are involved in the NF-κB signaling pathway induced by LPS but not by TNF-α or IL-1β; therefore, they are good candidates as molecular targets for immunomodulation mediated by *P. freudenreichii*-derived EVs with LPS-specific induction. Consequently, the abundances of their potential bacterial interacting partners might also explain the differences in EV activity as a function of culture medium. Among the predicted TLR4- and TICAM1-interacting bacterial proteins, six displayed a different abundance pattern between UF- and YEL-derived EVs. These included NifJ1, the NADH-quinone oxidoreductase chain G (NuoG), and an uncharacterized protein (PFCIRM129_01355), which were more abundant in UF-derived EVs, and GtfB glycosyltransferase (GtfB), a putative DNA polymerase I (PolA), and the UvrABC system protein A (UvrA3), which were more abundant in YEL-derived EVs. These proteins have not previously been characterized as being immunomodulatory, but our *in silico* network predictions suggest they can interact with human proteins with key roles in the inflammatory response; their abundances also could explain why UF- and YEL-derived EVs display distinct patterns of biological activity. Whether these proteins as well as other differentially abundant immunomodulatory proteins participate in the modulation of EV biological activity as a function of culture medium certainly deserves further investigation.

To sum up, our study showed that the probiotic *P. freudenreichii* CIRM-BIA129 produced EVs with distinct properties depending on the culture medium in which the bacterium was grown and reinforced the importance of environmental conditions to the properties of EVs. Although little is known about the mechanisms that underpin the modulation of Gram-positive bacterial EV production, content, and activity, they respond definitively to environmental stimuli in the case of *P. freudenreichii*. Specifically, in the context of probiotic bacteria, the optimization of medium composition might represent a tool to improve the beneficial activity of EVs and enable the development of EV-based therapeutic applications, such as functional foods with improved properties or pure EV formulations. Comprehensive studies might elucidate the relationships between specific medium components and the vesicular export of immunomodulatory proteins, enabling an improvement to the anti-inflammatory properties of EVs.

## MATERIALS AND METHODS

### Bacterial cultures.

*P. freudenreichii* CIRM-BIA129 (equivalent to the ITG P20 strain, provided by CNIEL) was supplied, stored, and maintained by the CIRM-BIA Biological Resource Center (Centre International de Ressources Microbiennes-Bactéries d'Intérêt Alimentaire, INRAE, Rennes, France). The bacteria were grown under two conditions: in yeast extract-lactate medium (YEL) or cow milk ultrafiltrate medium (UF), which was further supplemented with 100 mM sodium lactate and 5 g liter^−1^ casein hydrolysate, as previously described (63, 65). For both conditions, incubation was at 30°C, without agitation, until the start of stationary phase (2 × 10^9^ bacteria ml^−1^ for UF and 3 × 10^9^ bacteria ml^−1^ for YEL), as reported elsewhere (63). The culture starting volume was 500 ml for each biological replicate.

### Purification of EVs.

Bacterial cultures (500 ml) were centrifuged at room temperature (6,000 × *g*, 15 min) and the supernatants filtered through 0.22-µm top filters (Nalgene, Thermo Scientific). The supernatants were then concentrated 1,000-fold using 100-kDa ultrafiltration units (Amicon, Merck Millipore) by successive centrifugations (2,500 × *g*). The concentrated suspension of EVs was recovered in TBS buffer (Tris-buffered saline; 150 mM NaCl, 50 mM Tris-Cl, pH 7.5) and further purified by size exclusion chromatography (qEV original, 70 nm; iZON) as recommended by the manufacturer (30, 77). The resulting fractions containing EVs in TBS buffer were pooled (1.5 ml) and further concentrated to achieve approximately 50 µl using 10-kDa centrifugal filter units (Amicon, Merck Millipore). The aliquots were stored at −20°C or used immediately.

### Characterization of EVs.

The characterization of EVs in terms of size and concentration was performed by nanoparticle tracking analysis using a NanoSight NS300 (Malvern Panalytical), equipped with a sCMOS camera, Blue488 laser, and NTA 3.3 Dev Build 3.3.104 software. The experiments were conducted at 25°C, with a camera level of 15 and a syringe pump speed of 50. Concentration, focus, and other parameters were adjusted accordingly for each experimental group. A detection threshold of 3 was employed to determine valid particles. EV homogeneity and integrity were also confirmed by negative staining electron microscopy (data not shown).

### Evaluation of EV activity *in vitro*.

Two HT-29 human colon adenocarcinoma cell lines were employed to characterize the activity of EVs *in vitro*, as previously reported (30). Briefly, the parental HT-29 intestinal epithelial cells (ATCC HTB-38) were used to measure IL-8 release (IL-8/CXCL8 DuoSet; R&D Systems), and the lineage transfected with the secreted alkaline phosphatase (SEAP) reporter system (HT-29/kb-seap-25) was used to monitor NF-κB activity (Quanti-Blue reagent; Invivogen) (62). HT-29/kb-seap-25 cells were cultured in RPMI-glutamine medium (Sigma-Aldrich) supplemented with 10% fetal bovine serum (Corning), 1% nonessential amino acids, 1% sodium pyruvate, 1% HEPES buffer (Thermofisher Scientific), and 1% penicillin-streptomycin (Lonza) (62). The parental HT-29 cells were cultured in high-glucose Dulbecco’s modified Eagle medium (DMEM) (Dominique Dutscher) supplemented with 10% fetal bovine serum and 1% penicillin-streptomycin (76). The cells were treated with TBS buffer as a control or the EV preparations (1.0 × 10^9^ EVs ml^−1^) purified from UF or YEL cultures. To induce inflammation, the cells were also treated with TNF-α (1 ng ml^−1^; PeproTech), IL-1β (1 ng ml^−1^; Invivogen), LPS from Escherichia coli O111:B4 (1 ng ml^−1^; Sigma-Aldrich), or the TBS control. After seeding in 96-well plates (3 × 10^4^ cells/well) and stimulation with the samples, the cells were incubated at 37°C, under 5% CO_2_, for 24 h. Cell confluence was verified under the microscope before and after stimulation. Cell viability after stimulation was investigated using the CellTiter 96 AQueous one-solution cell proliferation assay (MTS; Promega). Absorbance was measured with a Xenius (SAFAS Monaco) microplate reader at 655 nm for the SEAP (NF-κB) activity assay, 450 nm for the IL-8 enzyme-linked immunosorbent assay, and 490 nm for the MTS cell viability assay.

### Identification and quantification of proteins using mass spectrometry.

One microgram of EV was dissolved in SDS and subjected to SDS-PAGE for a short period, allowing the entry of the protein into 2 to 3 mm of separating gel. The gel pieces were subjected to in-gel trypsinolysis followed by peptide extraction, as described previously (30, 72, 78). After digestion, the peptides were stored at −20°C until further analysis. Nano-liquid chromatography tandem mass spectrometry (LC-MS/MS) experiments were performed as previously described (30, 79). The peptides were identified from MS/MS spectra using X!TandemPipeline software (80), and searches were performed against the genome sequence of *P. freudenreichii* CIRM-BIA129 (GenBank accession no. NZ_HG975455). The database search parameters were specified as the following: trypsin cleavage was used, and peptide mass tolerance was set at 10 ppm for MS and 0.05 Da for MS/MS. Methionine oxidation was selected as a variable modification. For each peptide identified, an E value lower than 0.05 was considered a prerequisite for validation. A minimum of two peptides per protein was imposed, resulting in a false discovery rate (FDR) of <0.15% for protein identification. Each peptide identified by tandem mass spectrometry was quantified using the free MassChroQ software (81) before data treatment and statistical analysis with R software (R 3.4; Project for Statistical Computing). A specific R package, called MassChroqR (v0.4.3), was used to automatically filter dubious peptides and group the peptide quantification data into proteins. Two different and complementary analytical methods were used, based on peak counting or XIC (extracted ion current). For peak counting, variance analysis was performed on proteins with a minimum peak ratio of 1.5 between both culture conditions. Proteins with an adjusted *P* value of <0.05 were considered significantly different. For XIC-based quantifications, normalization was performed to take account of possible global quantitative variations between LC-MS runs. Peptides shared between different proteins were automatically excluded from the data set, as were peptides present in fewer than two of the three biological replicates. Missing data were then imputed from a linear regression based on other peptide intensities for the same protein. Analysis of variance was used to determine proteins whose abundance differed significantly between growth conditions.

### Proteomic analysis.

Protein subcellular localizations were predicted by Cello2GO (82); lipoproteins were predicted by PRED-LIPO (83) and Clusters of Orthologous Groups (COG) categories, and KEGG Pathways were predicted by eggNOG-mapper v2 (84, 85). The Venn diagram, heatmaps, and volcano plot were conceived using Python’s Matplotlib-venn 0.11.5, Seaborn 0.10.0, and Bioinfokit 0.7 packages, respectively.

### Functional enrichment analysis.

For functional enrichment analysis, the lists of up- and downregulated proteins were submitted to the g:Profiler web server (86, 87), together with an ortholog-based Gene Matrix Transposed (GMT) file (see Files S1 and S2 in the supplemental material) constructed from KEGG pathways and COG whole-proteome annotation categories obtained from eggNOG-mapper v2 (84, 85). Protein lists were ordered by decreasing relative level of abundance, and a significance threshold (adjusted *P* value) of 0.05 was adopted. For visualization, adjusted *P* values of 1 × 10^−6^ or lower were capped to this value.

### Prediction of protein-protein interactions.

Human and bacterial protein sequences were retrieved from the UniProt and NCBI databases, respectively. Bacterial sequences identified in EVs derived from at least one of the growth conditions, as well as the human sequences corresponding to KEGG’s NF-κB pathway, were submitted to the InterSPPI web server for the prediction of interactions (88). Only interactions with a score of 0.9765 or higher were considered valid, with a specificity of 0.99. These interactions were gradually filtered so that human counterparts corresponded to, or were specific to, the LPS-induced portion of the NF-κB pathway, and/or bacterial proteins displayed a significant fold change in their expression according to quantitative proteomics. The lists of human proteins used for filtering are available in Tables S1 to S3. Predicted interactions and protein annotations were processed using the Pandas Python package (89). Visualization of the interaction network was achieved using Cytoscape software (90).

### Statistical analysis.

All experiments were performed independently and at least in triplicate. The results are presented as means ± standard deviations. Absorbance measurements were normalized to the control conditions. The differences between experimental groups were analyzed using the Mann-Whitney test or two-way analysis of variance (ANOVA) followed by Tukey’s multiple-comparison test. Statistical analysis was performed using GraphPad Prism (GraphPad Software, San Diego, California, USA).

### Data availability.

The mass spectrometry proteomics data can be found at https://doi.org/10.15454/Q6PPXY.

## Supplementary Material

Supplemental file 1

Supplemental file 2
