# Supplementary material for: Environmental Conditions Modulate the Protein Content and Immunomodulatory Activity of Extracellular Vesicles Produced by the Probiotic Propionibacterium freudenreichii
Source: Appl Environ Microbiol. 2021 Jan 29;87(4):e02263-20. doi: 10.1128/AEM.02263-20 (PMC7851693; doi:10.1128/AEM.02263-20)
Supplement: Supplemental file 1 [file AEM.02263-20-s0001.pdf]

Supplementary Material

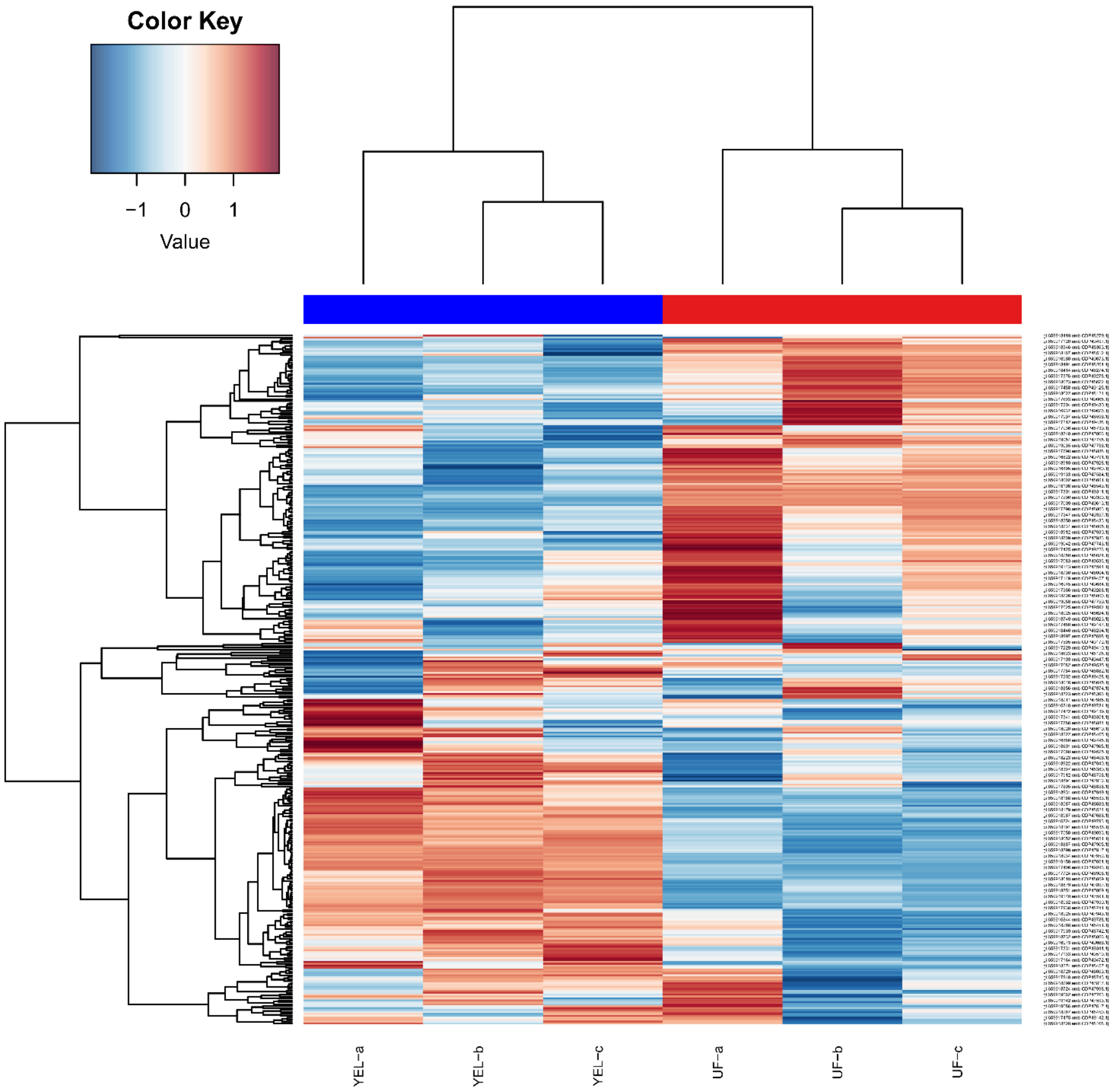

Figure S1. Heatmap representing variations in the relative abundance of identified proteins in biological replicates of YEL and UF-derived EVs.
